# Identification of copy number variations through whole genome resequencing between Jiuyishan and Hyplus rabbits

**DOI:** 10.3389/fvets.2025.1612883

**Published:** 2025-09-24

**Authors:** Yuanlang Wang, Jinzi Wang, Haisheng Ding, Lianxi Yang, Huiling Zhao, Dongwei Huang

**Affiliations:** ^1^Anhui Provincial Key Laboratory of Livestock and Poultry Product Safety, Institute of Animal Husbandry and Veterinary Medicine, Anhui Academy of Agricultural Sciences, Hefei, China; ^2^College of Animal Science and Technology, Hunan Agricultural University, Changsha, China

**Keywords:** Jiuyishan rabbit, Hyplus rabbit, whole genome resequencing, copy number variation (CNV), Vst

## Abstract

Copy number variations (CNVs), which include duplications and deletions of DNA segments, are significant structural variants that play crucial roles in the genetics of complex traits in livestock. High-throughput sequencing technologies enable the systematic identification of structural variants across genomes. However, CNV-based analyses of whole-genome sequencing data in rabbits remain largely unexplored. Herein, we characterized genome-wide CNVs of two rabbit breeds, Jiuyishan rabbit (JY) and Hyplus rabbit (HP), using whole-genome resequencing to elucidate their genetic characteristics and selection signatures. In total, 5,599 CNV regions (CNVRs) were identified between JY and HP, covering 0.98% of the reference genome. To identify selection signatures, we employed variance stabilizing transformation (Vst) values, selecting the top 1% of CNVRs with the highest Vst values, resulting in 56 CNVRs. These CNVRs harbored 27 genes. Functional analyses indicated that these genes were associated with important traits such as growth (*HOMER1*, *NOS1AP*, *PDE4B*, *LEPROT*) and reproduction (*FRAS1*, *CFAP43*, *TM9SF2*, and *CTNND2*). This study aims to enhance our understanding of CNVs and selection signals in rabbits, provide insights into the genetic differences between Chinese indigenous breeds and Western commercial lines, and offer valuable resources for investigating the genetic basis of complex traits.

## 1 Introduction

Modern rabbits (*Oryctolagus cuniculus*), known simply as rabbits, are among the most recently domesticated species, with domestication initiated in monasteries in southern France approximately 1,500 years ago (1, 2). This process was followed by artificial selection, leading to the establishment of numerous breeds. The resulting breeds can be distinguished based on their extensive phenotypic diversity, and over 300 recognized breeds have been identified worldwide (3). This diversity serves as an invaluable resource for genetic research and provides profound insights into the genetic mechanisms underlying phenotypic variation, disease resistance, and adaptation to different environments. Currently, China hosts at least 40 indigenous and recently introduced commercial rabbit breeds that are primarily distributed in provinces such as Shandong, Sichuan, Henan, and Hebei (4). Rabbits are widely used for meat, wool, and fur production. However, the Chinese meat rabbit industry relies heavily on foreign breeds and synthetic lines, and the development of superior domestic breeds is still limited (5). Jiuyishan rabbits (JY), an important indigenous genetic resource in Hunan Province, China, are renowned for their exceptional meat quality, high fertility, strong adaptability, and disease resistance, and are characterized by a relatively small body size and slow growth rate (5). Moreover, recently established rabbit lines or strains, such as Hyplus (HP), Hyla, and Hycole rabbits, have been developed through the cross-breeding of existing varieties or morphs. This process aims to introduce desirable traits and create novel combinations of morphological features, thereby enhancing production traits in specialized meat lines (6–8). Among these, Hyplus rabbits, developed by the French company Groupe Grimaud, encompasses eight special strains that were introduced to China in the early 21st century. They are characterized by rapid growth rate, high meat yield, and excellent reproductive performance (9). In recent years, the number of indigenous rabbit breeds has declined rapidly owing to the introduction of more efficient breeding programs and increased competition from foreign commercial breeds (5, 10). Nevertheless, considering the increasing recognition of local breeds as valuable genetic resources, several measures have been implemented to ensure their preservation.

Structural variation refers to genomic variations >1 kb, including copy number variations (CNVs), translocations, and inversions (11). CNVs, a specific type of structural variation typically ranging from approximately 50 bp to several 1 megabases, are primarily characterized by deletions and duplications (12). These variants can intersect with genes, altering their structure and expression, which in turn results in phenotypic variability and increased susceptibility to diseases in both humans (13, 14) and domestic animals (15–17). CNVs account for a significant proportion of heritability loss observed in genome-wide studies of certain traits. Although less prevalent than other molecular markers within the genome, CNVs encompass larger genomic regions and, consequently, exert significant effects on phenotypic variability (18). Unlike SNPs, CNVs span larger genomic regions and exhibit higher mutation rates, potentially having a more substantial impact on gene structure, regulation, and expression (19).

Several studies have explored the genetic basis of economically important traits and phenotypic variation in domestic rabbits. Indigenous Chinese rabbit breeds exhibit significant phenotypic diversity in coat color, body weight, body size, and meat quality, along with considerable genetic variation. However, information on CNVs in indigenous Chinese rabbits and their selection based on CNVs remain unexplored. Therefore, this study aimed to elucidate the genomic characteristics of JY and HP at the CNV level using whole-genome resequencing. This research will enhance our understanding of the physiology and genomic features of rabbits and provide a theoretical basis for the future breeding of native Chinese breeds.

## 2 Materials and methods

### 2.1 Sample collection and DNA extraction

**Figure 1:**
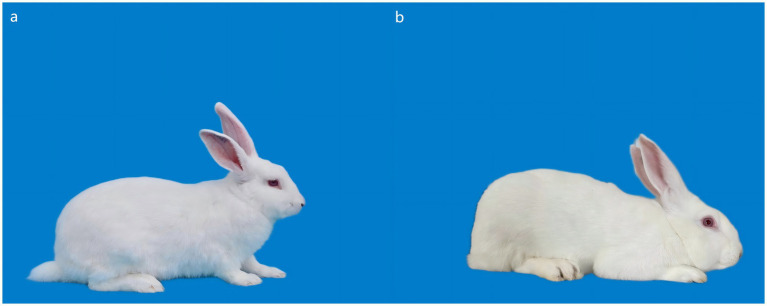
Picture of two breeds of rabbits: **(a)** Jiuyishan rabbit; **(b)** Hyplus rabbit.

Ear tissues from two rabbit populations (JY and HP; Figure 1) were collected from Hunan Hyplus Agriculture and Animal Husbandry Technology Co., Ltd. (Ningyuan County, Hunan Province, China). For genome sequencing, ear tissues were obtained from 28 male rabbits (13 JY and 15 HP). The sampled individuals had no direct or collateral blood relationships within the previous three generations. Genomic DNA was extracted using the standard phenol-chloroform extraction method to construct DNA sequencing libraries. Genomic DNA integrity and quality of were assessed using 0.5% agarose gel electrophoresis and a Nanodrop spectrophotometer (Thermo Fisher Scientific, Waltham, MA, United States).

### 2.2 Sequencing data generation and CNV calling

The sequencing libraries were prepared through a series of steps, including random DNA fragmentation, purification to obtain fragments of the desired length, adapter ligation, and DNA clustering. Sequencing was performed on the Illumina Hiseq X Ten (Illumina, San Diego, CA, United States) NGS platform at Gene Denovo Biotechnology Company (Guangzhou, China) generating 150 bp paired-end (PE150) read data. Each sample was sequenced at 10 × genome coverage. Quality control procedures were implemented to eliminate adapter contamination and low-quality reads based on the following criteria: (1) reads containing ≥10% unidentified nucleotides (N) were discarded; (2) reads where >50% of the bases had Phred quality scores ≤20 were excluded; and (3) reads aligning to barcode adapters were removed. The clean reads were aligned to the *Oryctolagus cuniculus* reference genome (Ensembl release 107) obtained from NCBI using the Burrows–Wheeler Aligner (BWA) software (version 0.7.19) (20) with default parameters. Subsequently, CNVcaller software was employed for CNV prediction relative to the OryCun 2.0 reference assembly (21). Firstly, the reference database was partitioned and overlapping windows of 800 bp were recommended. Second, the read counts in each window were calculated and reads with high similarity were merged into segments corresponding to autosomes. Third, GC bias was applied to normalize the copy number in each window, which facilitated the classification of different genotypes for each sample. Finally, the CNV calls were filtered using default parameters. CNVs were categorized as duplication, deletions and duplication-deletion CNVR regions (CNVR). The length of CNVR was defined as ≤50 kb for deletion and both types, while the length of CNVR for duplications was <500 kb.

### 2.3 Population differentiation

In the principal component analysis (PCA), the VCF files were converted into the MAP and PED formats using PLINK software (version 1.90). Subsequently, PCA was conducted using GCTA software (version 1.91) with default settings. TreeBest software (version 1.92) was employed to construct the evolutionary tree, which was subsequently visualized using ITOL v7^1^ (22) and a genomic relationship matrix was constructed used GEMMA (R package) with -gk and default option to estimate the kinship matrix.

### 2.4 Sweep selective analysis of the CNVR

The V_ST_ parameter between JY and HP was calculated to identify differential CNVRs.

The V_ST_ operates on principles similar to the F_ST_ statistic, a well-established measure for evaluating genetic differentiation between populations, and provides an unbiased measure of the F_ST_. However, the V_ST_ is tailored to quantify population differences based on copy number variation data. The formula for V_ST_ is: $V_{\mathrm{ST}}=(V_{\text{total}}-(V_{\mathrm{pop}1}\times N_{\mathrm{pop}1}+V_{\mathrm{pop}2}\times N_{\mathrm{pop}2})/N_{\text{total}})/V_{\text{total}}$ , where V_total_ represents the total variance in the copy number between the two groups, V_pop1_ and V_pop2_ are variances in the copy numbers within population 1 and population 2, respectively. N_pop1_ and N_pop2_ represent the sample sizes of populations 1 and 2, respectively. And N_total_ is the total sample size. Subsequently, the top 1% of areas, defined as those having received strong selection, were identified. The CNVRs with the top 1% V_ST_ values were then selected as candidate regions for further analysis.

Functional enrichment analysis of these CNVRs was conducted using ANNOVAR for annotation. Additionally, Gene Ontology (GO) and Kyoto Encyclopedia of Genes and Genomes (KEGG) pathway analyses were performed on the candidate CNV-associated genes using KOBAS 3.0^2^ (23). FDR was used to adjust the *p*-value, and the critical value of the adjusted *p*-value with a significance threshold of <0.05.

## 3 Results

### 3.1 Identification of CNVR

**Figure 2:**
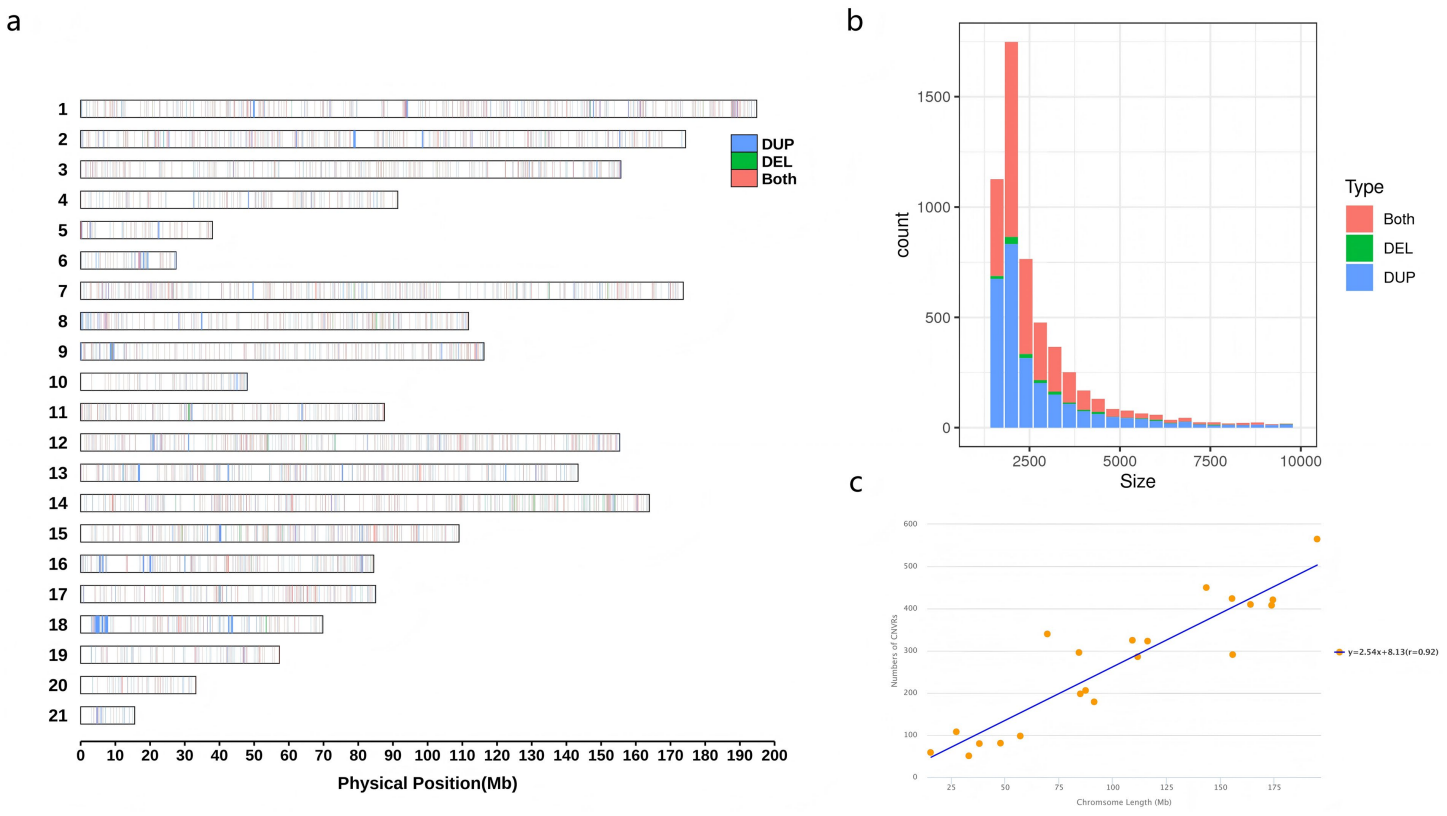
Genomic diversity and distribution of CNVRs. **(a)** Distribution of CNVRs on chromosome ideogram according to their state. The CNVRs are illustrated in green (delections), blue (duplications) and red (both of delections and duplications). **(b)** Distribution of CNVRs size by state. **(c)** Correlation between CNVR counts and chromosome length.

Whole-genome sequencing of 13 JY and 15 HP rabbits was conducted to detect genome-wide CNVRs and to compare the differentiation between the two populations. The mapped read depth ranged from 10.07 × to 12.99×, with an average depth of 10.77 × per sample, and the average mapping rate was 98.30% (Supplementary Table S1). These results indicated that the data were of sufficient quality for further analysis. A total of 5,599 CNVRs were obtained, with an average length of 3775.39 bp, covering 0.99% of the reference genome. These included 2,771 duplication CNVRs, 138 deletion CNVRs, and 2,690 duplication-deletion CNVRs (Supplementary Table S2). A total of 565 CNVR identified on the largest chromosome (chromosome 1) and 59 CNVRs for the smallest chromosome (chromosome 21) (Figure 2a). The sizes of all CNVRs showed an L-shaped distribution, which was detected in 1.6–282 kb, with approximately 3,018 CNVRs (53.90%) between 2 and 5 kb (Figure 2b). There was a significant positive linear correlation between the number of CNVRs and the corresponding chromosome length (*r* = 0.92, Figure 2c).

### 3.2 Population structure analysis

**Figure 3:**
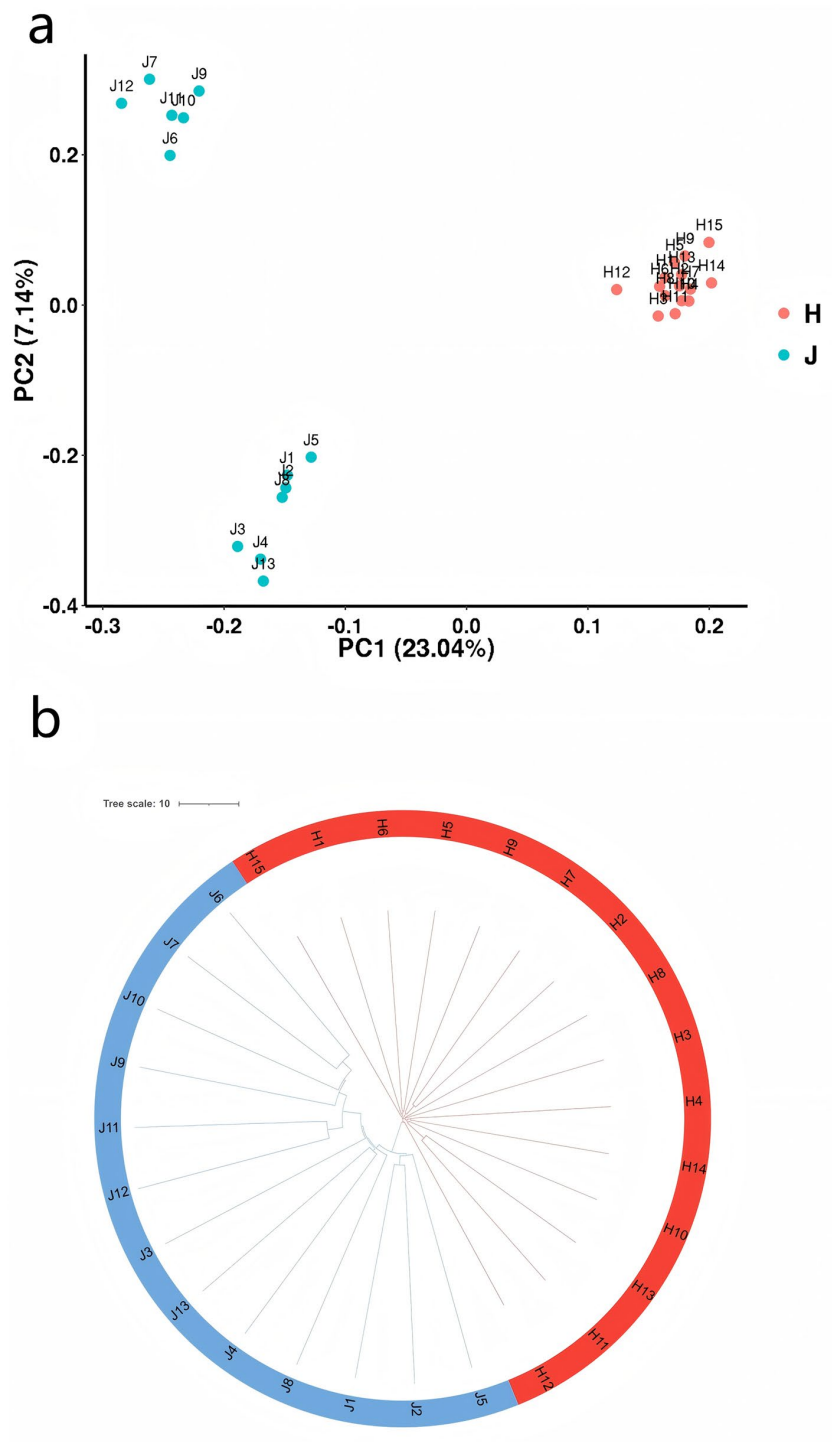
Analysis of population structure of two rabbit breeds. **(a)** PCA derived from all CNVRs in two population genomes. Red nodes and blue nodes represent the Hyplus and Jiuyishan rabbit groups, respectively. **(b)** Evolutionary tree constructed from CNVRs. Red stripes and blue stripes represent the Hyplus and Jiuyishan rabbit groups, respectively.

The heatmap of genomic relationships between individuals, as shown in Supplementary Figure S1, indicates that individuals from each samples are not clustered together. Principal component analysis (PCA) was carried out to distinguish reproducible differences between JY and HP populations (Figure 3a). The first two principal components (PC1: 23.04%, PC2: 7.14%) could separate JY and HP breeds. To verify the repeatability of the samples from these two populations, genetic distances among individual samples were calculated using an evolutionary tree (Figure 3b). The results showed that CNVR clustering was readily divided into two branches. This clustering-based approach corroborated the findings of the PCA. Interestingly, the JY population exhibited greater genetic separation within these breeds owing to duplication events. In contrast to the HP rabbits, the JY rabbits may have experienced less selective pressure, leading to numerous nonfunctional duplications.

### 3.3 Differentiated CNVRs between JY and HP

**Figure 4:**
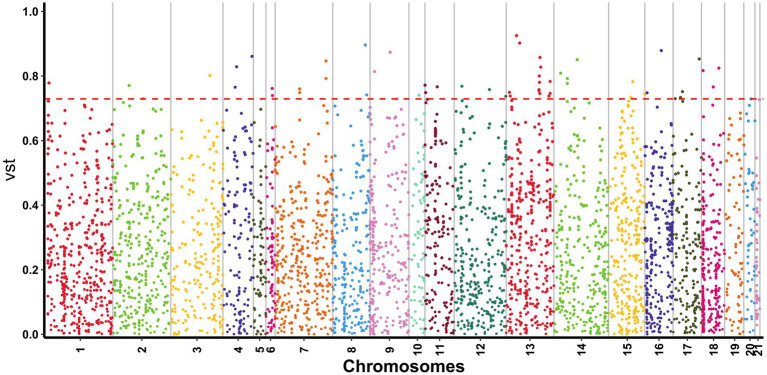
Manhattan plot of genome-wide Vst on each CNVR locus between HP and JY. The red line represents the threshold line of top 1% of Vst. Points located above the red line were identified as selected CNVRs.

**Figure 5:**
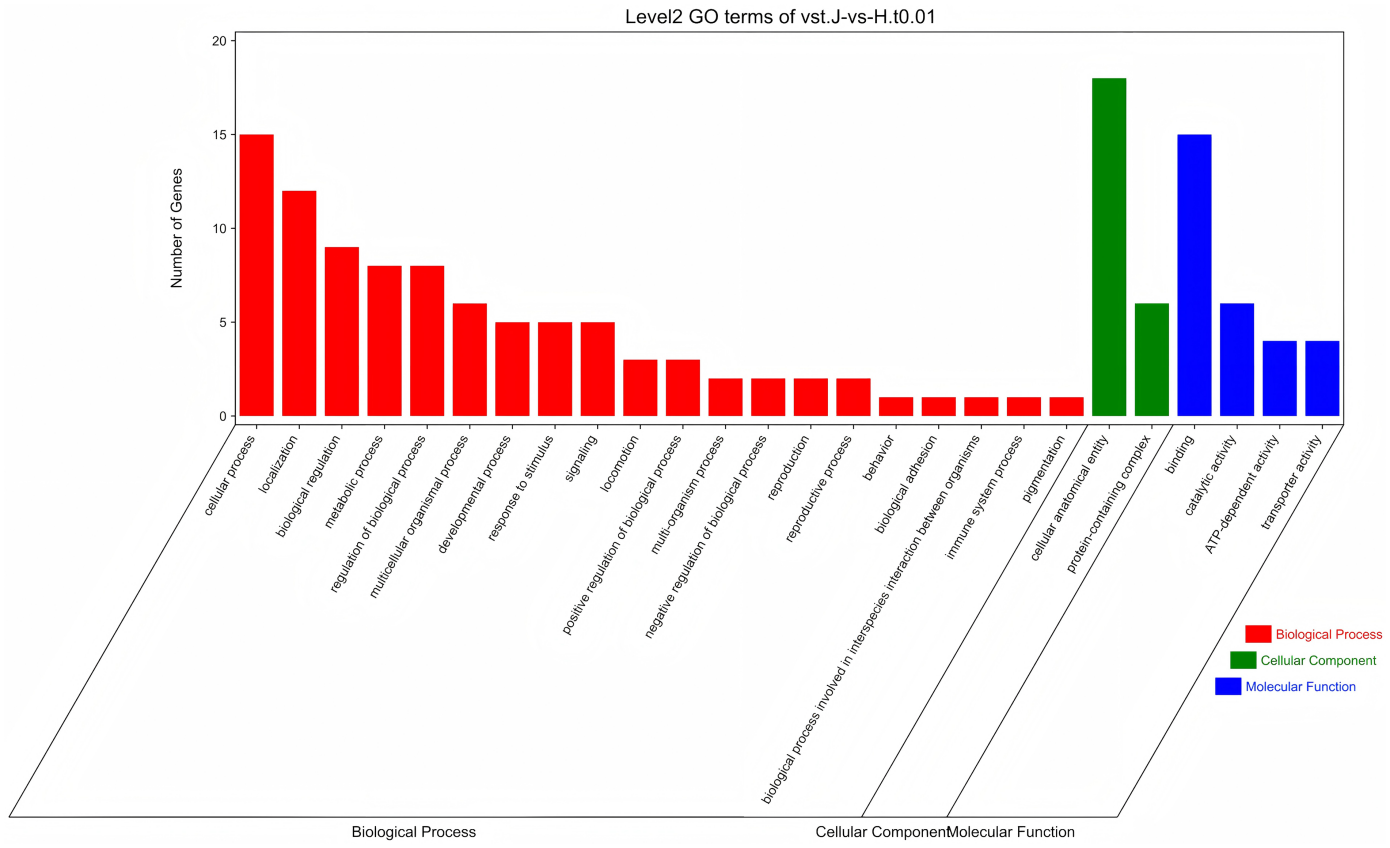
GO enrichment analysis of the select genes. Red referring to biological process, green referring to cellular component, and blue referring to molecular function.

**Figure 6:**
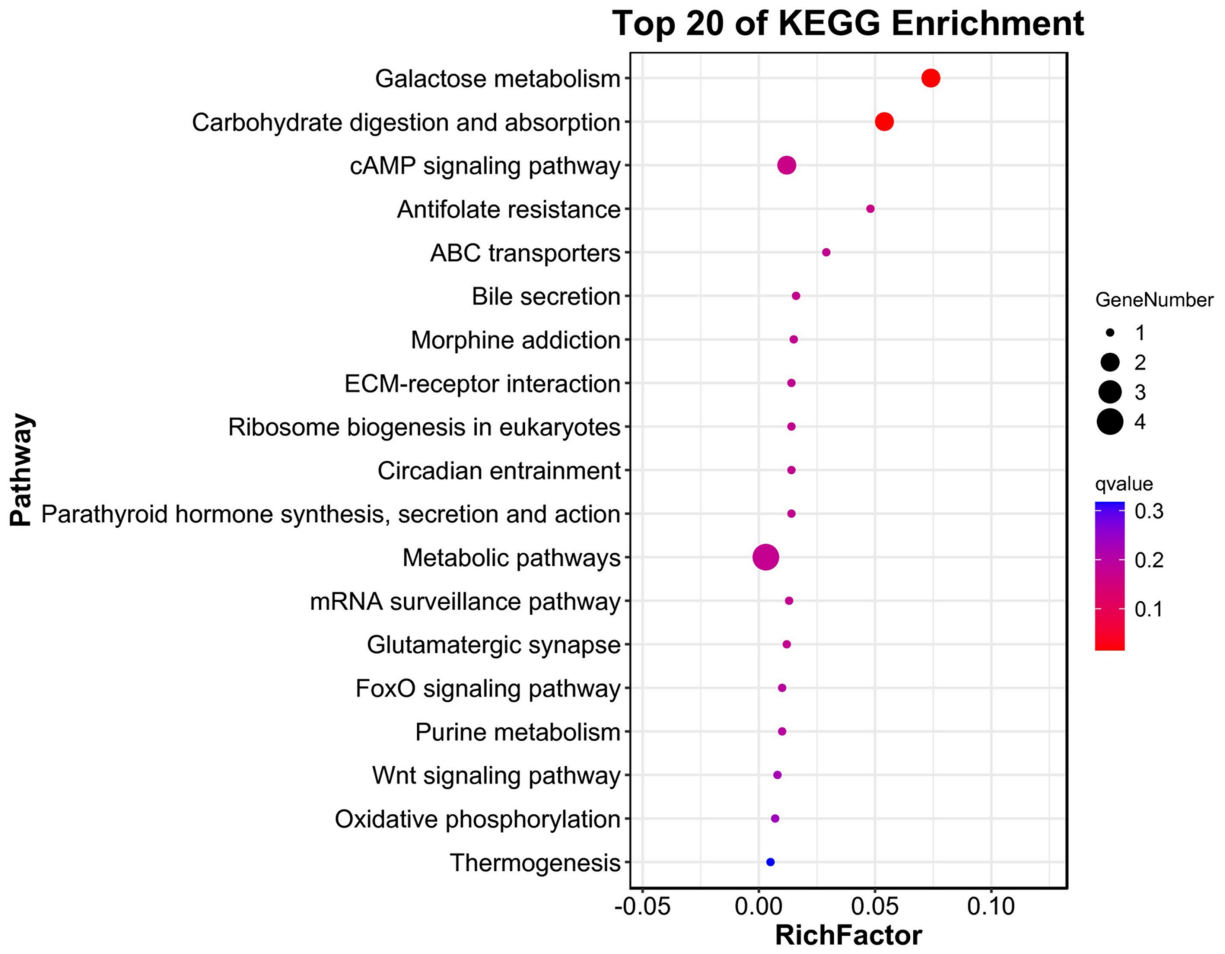
KEGG analysis of the selected genes. The size of dot refers to the number genes related to pathway, and the red to blue indicate the significant value of adjusted *p* change.

All CNVRs were compared to the rabbit genome database, and Vst analysis was performed for each CNVR-related gene to identify population-specific selection signatures. The Manhattan plot shows the results of Vst with chromosomes in the horizontal coordinates and Vst values in vertical coordinates (Figure 4). Identifying genes with high differentiation between different breeds. The top 0.01 was taken as the threshold line (threshold: 1%, Vst = 0.7335). The results showed that 56 outlier loci that, overlapped with 27 genes, exceeded the threshold in the two breeds (Supplementary Table S3). To better understand genes with a high degree of variation among varieties. All genes were identified and carried out to GO enrichment and KEGG pathways analyses. A total of 26 GO terms were enriched at level 2 GO enrichment, which included cellular processes (GO:0009987), binding (GO:0005488), biological regulation (GO:0065007), catalytic activity (GO:0003824), response to stimulus (GO:0050896), and ATP-dependent activity (GO:0140657) (Figure 5 and Supplementary Table S4). KEGG analysis revealed 19 pathways, including galactose metabolism (ko00052), carbohydrate digestion and absorption (ko04973), cAMP signaling pathway (ko02010), ECM-receptor interaction (ko04512), and Fox0 signaling pathway (ko04068) (Figure 6 and Supplementary Table S5). Among them, galactose metabolism, carbohydrate digestion, and absorption were significantly enriched.

## 4 Discussion

Domestication, followed by directional artificial and natural selection, as well as several other genetic events, has shaped the genomes of domestic animals, resulting in differentiation of numerous breeds and populations within species (24). CNVs are crucial sources of genetic diversity and show variations among animal breeds. Some unique CNVs may have been selected during domestication, contributing to species-specific traits. In recent years, CNVs have been widely used as a supplementary tool in association studies, aiding in the identification of genetic variants associated with economically important traits and elucidating the genetic basis of these traits across different livestock species (25). However, studies on rabbit CNVs are limited.

In this study, we identified 5,599 CNVRs in two rabbit species using the whole-genome resequencing (WGRS) technique. Compared to previous studies based on array comparative genome hybridization (26), we detected more than 30 times more CNVRs per rabbit population. Additionally, our results demonstrate that WGRS and long-read sequencing provide superior precision for breakpoint identification, enhanced sensitivity, and higher resolution than array-based technologies. Among the identified CNVRs, a positive linear correlation was observed between the number of CNVRs and chromosomes length. Additionally, the number of duplication events exceeded that of deletions, a pattern consistent with findings reported previously (27, 28). The inconsistencies in CNVR counts observed across studies may be attributed to differences in sample sizes, detection methods, and reference genomes. Consequently, most newly identified CNVRs can be considered novel, thereby enriching existing research on CNVs in rabbits. This is the first comprehensive investigation of genomic CNVR maps across different rabbit populations.

Genes located within CNVRs with diverse molecular functions serve as valuable resources for elucidating the biological relationships between CNVRs and the genetic basis of phenotypic variation. GO enrichment analysis revealed that the detected CNVR-overlapping genes in both rabbit breeds were significantly enriched in reproductive, immune system, and developmental processes. The enrichment of functional categories among CNV-harboring genes likely reflects targeted artificial selection of physical traits in rabbits. Under such selective pressures, CNVs may accumulate in rabbit breeds, thereby forming a genetic basis for important economic traits. A valuable finding from KEGG pathway analysis was the cAMP signaling pathway, which plays a pivotal role in coordinating complex cellular processes, such as cellular growth and proliferation, differentiation, hormone production, and secretion (29). As a secondary messenger, cAMP regulates a broad spectrum of cellular functions in response to the extracellular stimuli. Localized cAMP signaling elicits distinct cellular responses. Studies have demonstrated that the cAMP signaling pathway is crucial for regulating adipogenesis in both mice and rabbits (30). Furthermore, evidence suggests its involvement in mammalian ovarian function (31).

Regarding growth traits, several genes (*HOMER1*, *NOS1AP*, *PDE4B*, and *LEPROT*) have been associated with fatty acid metabolism and skeletal muscle development. *HOMER1* encodes a scaffold protein primarily localized in postsynaptic structures, where it interacts with metabolic glutamate receptors (32). Studies have shown that *HOMER1* is involved in skeletal muscle contraction, development of skeletal muscle fibers, and regulation of Ca^2+^ channel activity (33). Nitric oxide synthase 1 adaptor protein (*NOS1AP*) has been reported to be linked to fatty acid metabolism in cattle (34). Additional studies indicated that *NOS1AP* plays a critical role in regulating hepatic insulin sensitivity and p38 MAPK inactivation in obese mice, highlighting its potential as a therapeutic target for type 2 diabetes prevention and treatment (35). *PDE4B*, a member of the PDE4 family that specifically hydrolyzes intracellular cAMP, has been implicated in fat deposition and may serve as a positional candidate gene for intramuscular fat in pigs (36). The leptin receptor overlapping transcript (*LEPROT*) has been suggested to play multiple roles in immune system regulation (37). Furthermore, studies have demonstrated that *LEPROT* is associated with fat synthesis and body weight in pigs (38).

In terms of reproductive traits, several notable genes were identified, including *FRAS1*, *CFAP43*, *TM9SF2*, and *CTNND2*. *FRAS1* encodes an extracellular matrix protein that plays a critical role in epidermal basement membrane formation during gestation (39). Mutations in this gene have been linked to Fraser syndrome in humans (40), whereas polymorphisms in *FRAS1* are highly expressed in the oviducts of pigs and can serve as biomarkers of sow fertility (41). Cilia- and flagella-associated protein 43 (*CFAP43*) has been implicated in multiple morphological abnormalities of sperm flagella. Recent studies have reported that CFAP43-null male mice exhibit infertility due to defects in the sperm flagellar structure (42, 43). Furthermore, significant correlations between CFAP43 expression levels and litter size in goats have been reported (44). A previous study also identified CNV in CFAP43 of domestic pigs that were associated with reproductive traits (45). *TM9SF2*, a member of a highly conserved transmembrane 9 protein superfamily characterized by nine putative transmembrane domains, has been identified as a candidate gene associated with sperm morphology and distal midpiece reflex in a Duroc boar populations (46). The *CTNND2* gene encodes delta-catenin protein, a key component of the adherens junction complex, and plays an important role in neuronal structure and function. Studies have demonstrated that *CTNND2* participates in the regulation of cell proliferation and influences the number of body segments in zebrafish (47). Additionally, prior research has established a connection between *CTNND2* and pig litter traits, as well as body size (48, 49). Notably, *CTNND2* plays an essential role in retinal morphogenesis, adhesion, and the architectural integrity of retinal cells in animal models (50). Based on these findings, it is reasonable to hypothesize that CNV events involving *CTNND2* are involved in the regulation of retinogenesis in domestic rabbits.

Given these critical functions, the genes harboring CNV identified in this study emerge as promising molecular markers, which hold significant potential for guiding future breeding strategies aimed at enhancing indigenous Chinese rabbit breeds, these findings provide a genetic basis for targeted improvements in traits relevant to livestock production and health.

While this genome-wide CNV profiling of the two rabbit populations provides valuable structural variation data, certain limitations remain and should not be overlooked. The lack of orthogonal biological validation—such as qPCR confirmation for high-impact CNVRs—introduces uncertainty regarding the accuracy of boundary delineation, especially for complex tandem duplications. In future studies, we anticipate that additional high-quality genome assemblies representing diverse populations, along with experimental validation, will enhance our understanding of the evolutionary mechanisms underlying CNV in rabbits.

## 5 Conclusion

In this study, we developed the first resequencing-based CNV map for rabbits. We investigated the genomic characteristics and structural variations between Jiuyishan rabbits and Hyplus rabbits and identified 5,599 CNVRs. Among these, several candidate genes were identified as potentially contributing to growth traits (*HOMER1*, *NOS1AP*, *PDE4B*, and *LEPROT*) and reproductive traits (*FRAS1*, *CFAP43*, *TM9SF2*, and *CTNND2*). These findings further illustrate genetic adaptations to ecological niches and management practices, offering valuable insights for precision breeding strategies aimed at improving productivity. Although further biological validation is necessary, our findings offer significant insights into the molecular mechanisms underlying key phenotypic variations in rabbits. Additionally, these results will facilitate future selection and improvement of economically important traits in native Chinese rabbit breeds.

## Data Availability

The datasets presented in this study can be found in online repositories. The names of the repository/repositories and accession numbers can be found in the Supplementary Table S1.

## Footnotes

^1^ https://itol.embl.de/

^2^ http://bioinfo.org/kobas/
