# Supplementary material for: Identification of copy number variations through whole genome resequencing between Jiuyishan and Hyplus rabbits
Source: Front Vet Sci. 2025 Sep 24;12:1612883. doi: 10.3389/fvets.2025.1612883 (PMC12504071; doi:10.3389/fvets.2025.1612883)
Supplement: Supplementary file 2 [file Image_1.pdf]

Figure supplementary 1

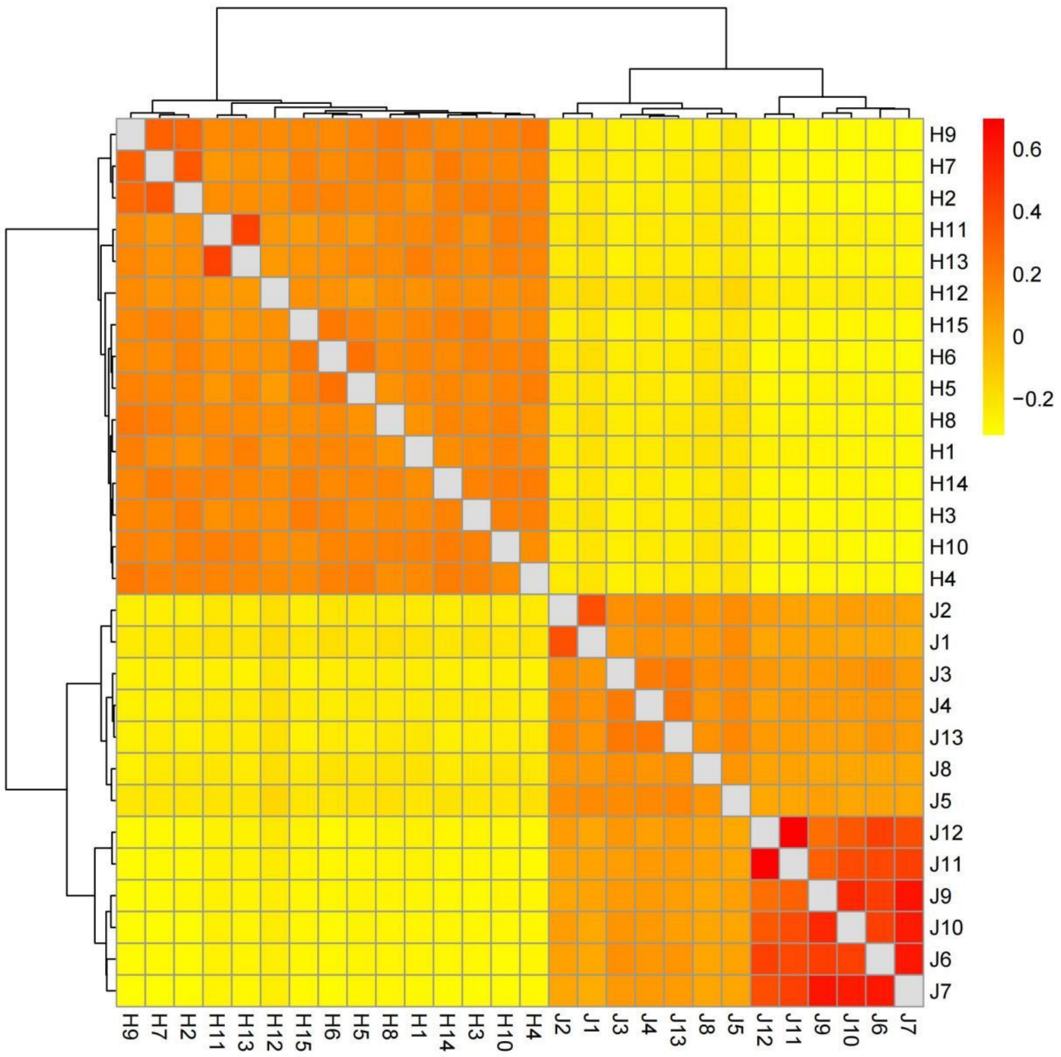

Heatmap of all 28 sequenced individuals using the molecular relationship matrix. The exact genomic relationship between two individuals is shown in each small lattice. The larger the value within 0-1, the closer the kinship.
